# HLA class II gene associations in African American Type 1 diabetes reveal a protective HLA-DRB1*03 haplotype

**DOI:** 10.1111/dme.12148

**Published:** 2013-03-21

**Authors:** J M M Howson, M S Roy, L Zeitels, H Stevens, J A Todd

**Affiliations:** 1 JDRF/Wellcome Trust Diabetes and Inflammation Laboratory, NIHR Biomedical Research Centre, Department of Medical Genetics, Cambridge Institute for Medical Research, University of CambridgeCambridge, UK; 2 Department of Public Health and Primary Care, University of Cambridge, Strangeways Research LaboratoryCambridge, UK; 3 Institute of Ophthalmology and Visual Science, University of Medicine and Dentistry of New JerseyNewark, NJ, USA

## Abstract

**Aims:**

Owing to strong linkage disequilibrium between markers, pinpointing disease associations within genetic regions is difficult in European ancestral populations, most notably the very strong association of the HLA-DRB1*03-DQA1*05:01-DQB1*02:01 haplotype with Type 1 diabetes risk, which is assumed to be because of a combination of *HLA-DRB1* and *HLA-DQB1*. In contrast, populations of African ancestry have greater haplotype diversity, offering the possibility of narrowing down regions and strengthening support for a particular gene in a region being causal. We aimed to study the human leukocyte antigen (HLA) region in African American Type 1 diabetes.

**Methods:**

Two hundred and twenty-seven African American patients with Type 1 diabetes and 471 African American control subjects were tested for association at the HLA class II genes, *HLA-DRB1*, *HLA-DQA1*, *HLA-DQB1* and 5147 single nucleotide polymorphisms across the major histocompatibility complex region using logistic regression models. Population admixture was accounted for with principal components analysis.

**Results:**

Single nucleotide polymorphism marker associations were explained by the HLA associations, with the major peak over the class II loci. The HLA association overall was extremely strong, as expected for Type 1 diabetes, even in African Americans in whom diabetes diagnosis is heterogeneous. In addition, there were unique features: the HLA-DRB1*03 haplotype was split into HLA-DRB1*03:01, which confers greatest susceptibility in these samples (odds ratio 3.17, 95% CI 1.72–5.83) and HLA-DRB1*03:02, an allele rarely observed in Europeans, which confers the greatest protection in these African American samples (odds ratio 0.22, 95% CI 0.09–0.55).

**Conclusions:**

The unique diversity of the African HLA region we have uncovered supports a specific and major role for *HLA-DRB1* in HLA-DRB1*03 haplotype-associated Type 1 diabetes risk.

## Introduction

Owing to genome-wide single nucleotide polymorphism (SNP) association studies in Type 1 diabetes, over 50 Type 1 diabetes susceptibility regions have now been reported (http://www.t1dbase.org) 1–4. However, because of the strong linkage disequilibrium between markers, and relatively low haplotype diversity, narrowing down disease-associated regions to aid candidate gene selection is often difficult in European ancestral populations. African ancestral populations have much greater haplotype diversity, offering the possibility of providing support for a smaller genomic region containing the causal variant(s) and, in some instances, a particular causal candidate gene. This approach was first described explicitly in Type 1 diabetes in a human leukocyte antigen (HLA) study of 37 patients (cases) and 79 controls ascertained from Afro-Caribbean communities in England by Todd *et al*. 5 and later confirmed in 98 African American patients 6. Both studies reported an African-specific HLA-DRB1*07- HLA-DQB1*02 haplotype that predisposed to Type 1 diabetes and carried the 03:01 allele at *HLA-DQA1*, which is associated with increased risk of Type 1 diabetes in Europeans, suggesting a causal role for *HLA-DQA1* 5. However, these studies, and subsequent African Type 1 diabetes reports, did not control for the complex admixture that can be extreme in populations such as African Americans 7–9. Furthermore, despite these efforts and genetic analyses of very large European populations, including the application of dense SNP marker maps of the major histocompatibility complex (MHC) region that mapped susceptibility to the *HLA-DQB1*, *HLA-DRB1*, *HLA-B* and *HLA-A* genes 10,11, the specific HLA genes responsible for the high predisposing effects of HLA-DRB1*03 haplotypes remain uncertain. It is assumed that *HLA-DRB1* and *HLA-DQB1* are major determinants.

**What's new?**

- This is the largest study in African Americans with Type 1 diabetes to date and the only study to account for ancestry.
- This is the only study to date that fine maps the HLA region in African American samples using a dense map of 5147 single nucleotide polymorphisms and the class II genes.
- We find HLA Type 1 diabetes associations that agree with known associations in Europeans, and novel associations of African ancestry specific alleles, e.g. HLA-DRB1*03:02, which confers protection from Type 1 diabetes.

Here, we typed African American samples with the dense immune disease SNP chip, ImmunoChip, with over 5000 SNPs in the HLA region 12 (http://www.immunobase.org), performed classical HLA class II typing, and adjusted for ancestry using principal component analysis. We obtained evidence for a specific role for the *HLA-DRB1* gene on HLA-DRB1*03 haplotypes.

## Subjects and methods

### Subjects

In total, 227 subjects with clinically defined Type 1 diabetes were recruited from among 13 615 African Americans discharged from 31 New Jersey hospitals between 1982 and 1996 13. All patients had acute onset of disease, for which they were admitted to hospital. A full description of the sample collection can be found in Roy 13. Diagnosis was confirmed through a documented elevated random or postprandial venous plasma glucose level > 11.1 mmol/l and an elevated fasting glucose > 7.8 mmol/l. Over 12 000 samples were excluded from the collection because of the subjects having Type 2 diabetes, or having no initial need of insulin therapy, or because they were over 30 years of age at diagnosis. All subjects were taking insulin, had very low or no detectable C-peptide, and were under 30 years of age at diagnosis (median 17 years). The 497 African American control subjects were sex-matched to case subjects, were of all ages, and were recruited from local community organizations, blood bank attendees and medical clinic attendees in New Jersey, had no diabetes and no family history of diabetes.

All DNA samples were collected after approval from the University of Medicine and Dentistry of New Jersey Institutional Review Board research ethics committee and written informed consent was obtained from the participants.

### Genotyping

Samples were genotyped using an Illumina 200K Infinium high-density array, Immunochip (http://www.immunobase.org) 12 according to the manufacturer's protocol at the Wellcome Trust Sanger Institute, Hinxton, UK. The SNP chip comprised 195 806 SNPs and 718 insertions/deletions covering 186 confirmed autoimmune disease-associated regions. SNPs with call rate below 0.95 or out of Hardy–Weinberg equilibrium with *P* < 1 × 10^–6^ were excluded. Owing to the small sample size compared with studies of Europeans, only SNPs with a minor allele frequency > 0.1 outside of the MHC region and 0.05 within the MHC region were included. Checks for sample heterozygosity, duplicate samples, related samples, inconsistent sex information and sample call rate < 0.9 were used to exclude samples. Samples with excessive European or Asian proportions from principal component analysis were removed (see also Supporting Information, Figs S1, S2 and S3). After all quality control measures, 227 case samples and 471 control samples were retained.

The HLA class II genes were genotyped using the LABType® SSO which applies Luminex® technology to the reverse sequence-specific oligonucleotide (SSO) DNA typing method.

### Statistical methods

SNP chip quality control was performed using the snpStats library (http://www.bioconductor.org) in R (http://www.r-project.org).

SNPs and genes were modelled (and odds ratios calculated) using logistic regression with disease status as outcome variable and allele(s), assuming multiplicative allelic effects, as independent variable(s). The first three principal components were included as confounders and a likelihood ratio test employed to test for association of the allele(s) with Type 1 diabetes. Association analyses were performed with STATA version 10 (http://www.stata.com). Power calculations were performed using QUANTO (http://hydra.usc.edu/GxE/). Haplotypes were reconstructed under the alternative (separately in cases and controls) using an expectation–maximization (EM) algorithm as implemented in the R library, haplo.stats (http://www.r-project.org).

## Results

Our study had 80% power to detect an odds ratio of 3.6 at genome-wide significance, α = 5 × 10^–8^, and an odds ratio of 2.5 at α = 0.001, assuming a minor allele frequency of 0.05 and a multiplicative allelic effects model. With the sample sizes currently available, analysis of small subgroups creates non-replicable false positive results owing to low statistical power. Therefore, although the HLA class II genes were genotyped to four-digit resolution, to maximize statistical power, we mostly analysed and report two-digit alleles at *HLA-DRB1* and *HLA-DQB1*, unless four-digit alleles were sufficiently common. There are fewer *HLA-DQA1* four-digit alleles, hence, this locus was analysed at four-digit resolution.

### Single-locus association

**FIGURE 1:**
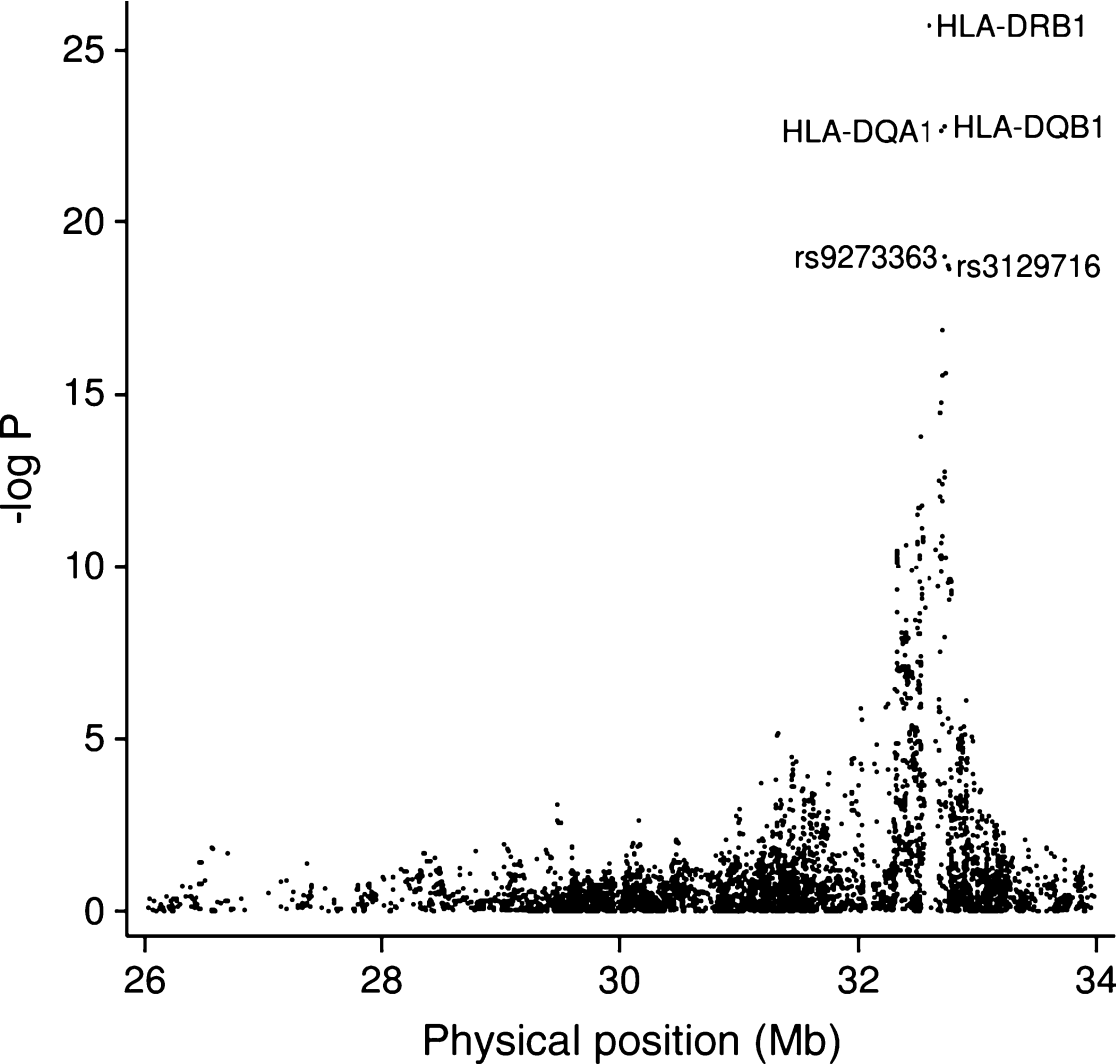
Association of 5147 single nucleotide polymorphisms (SNPs) and three genes across the extended major histocompatibility complex (MHC) region in up to 227 African American type 1 diabetes and 471 controls. All analyses were adjusted for population ancestry using the first three principal components, and signal clouds were manually checked for SNPs discussed in the manuscript with positive associations. Note, the minor A allele at rs9273363, the most associated SNP, was in linkage disequilibrium with the highly susceptible HLA-DRB1*03:01-HLA-DQA1*05:01-HLA-DQB1*02:01 (*r*^2^ = 0.43, *D*' = 1.00) and HLA-DRB1*04.HLA-DQA1*03:01-HLA-DQB1*03:02 haplotypes (*r*^2^ = 0.23, *D*' = 1.00).

The most convincing evidence of association in the MHC region was in the HLA class II region (Fig. 1). All three HLA class II genes tested were associated with Type 1 diabetes in these African American samples. *HLA-DRB1* was the most associated gene (*P* = 7.79 × 10^–26^) and rs9273363C>A the most associated SNP (*P* = 9.72 × 10^–20^; situated ∼1 kb 3′ of *HLA-DQB1*). This SNP had less evidence of association than *HLA-DQB1* (*P* = 1.62 × 10^–23^) or *HLA-DQA1* (*P* = 2.13 × 10^–23^), particularly given that the HLA gene association tests are on more degrees of freedom, which reduces power.

### Specific HLA class II alleles

While the general association pattern across the region is consistent with that reported in European samples 10,11, the nature of the HLA class II gene associations in African American samples differed to those in European samples. Most informatively, the highly susceptible haplotype in Europeans, signified by HLA-DRB1*03 14, is split in African Americans, into two haplotypes, one with a *HLA-DRB1* allele that confers the greatest disease susceptibility in our study, HLA-DRB1*03:01 (odds ratio 3.17, 95% CI 1.72–5.83; Table 1) and the other with the allele that confers greatest protection, HLA-DRB1*03:02 (odds ratio 0.22, 95% CI 0.09–0.55). The risk allele, HLA-DRB1*03:01 has a frequency of approximately 0.14 in British control subjects (see also Supporting Information, Table S1; 15) and 0.05 in African American control subjects. In contrast, the HLA-DRB1*03:02 allele, which is rarely detected in Europeans, has a frequency of 0.07 in African Americans. This allele is in linkage disequilibrium with rs6927077 and rs6940690 (*r*^2^ = 0.79, *D*' = 0.91 in control subjects), which were themselves associated with Type 1 diabetes in African Americans (*P* < 5 × 10^–5^, odds ratio 0.26, 95% CI 0.12–0.55) but, remarkably, are monomorphic in the European ancestry CEPH samples and British control subjects (http://www.t1dbase.org/page/Overview/display/marker_id/6927077; http://www.t1dbase.org/page/Overview/display/marker_id/6940690), yet had a minor allele frequency of 0.07 in the African ancestry Yoruban (YRI) population.

**Table 1:** Allele frequencies and association results for *HLA-DRB1* in 227 African American cases of Type 1 diabetes and 471 controls

	Allele frequency, *n* (%)				
				
*HLA-DRB1* allele	Cases	Controls	Odds ratio [95% CI]‡	Odds ratio [95% CI]‡	*P*_T1D_
03:01	85 (18.7)	51 (5.4)	3.17 [1.72–5.83]	4.19 [2.56–6.88]	
04*	74 (16.3)	58 (6.2)	2.37 [1.27–4.42]	3.13 [1.87–5.23]	
09:01	26 (5.7)	21 (2.2)	2.35 [1.09–5.09]	3.11 [1.55–6.27]	
07:01	59 (13.0)	81 (8.6)	1.63 [0.89–2.99]	2.15 [1.32–3.52]	
01	26 (5.7)	59 (6.3)	1.00 (reference)	1.32 [0.74–2.37]	
08	29 (6.4)	67 (7.1)	0.86 [0.43–1.74]	1.14 [0.66–1.99]	
13	60 (13.2)	163 (17.3)	0.76 [0.42–1.36]	1.00 (reference)	
11	35 (7.7)	135 (14.3)	0.51 [0.28–0.96]	0.68 [0.41–1.12]	
15:03	20 (4.4)	109 (11.6)	0.39 [0.19–0.80]	0.52 [0.29–0.94]	
15:01	5 (1.1)	23 (2.4)	0.36 [0.11–1.11]	0.47 [0.16–1.36]	
12	7 (1.5)	43 (4.6)	0.36 [0.13–0.94]	0.47 [0.19–1.14]	
03:02	8 (1.8)	66 (7.0)	0.22 [0.09–0.55]	0.29 [0.13–0.67]	
Rares†	20 (4.4)	66 (7.0)	0.68 [0.32–1.42]	0.90 [0.48–1.69]	
16	5 (1.1)	14 (1.5)			
10:01	10 (2.2)	29 (3.1)			
14	3 (0.7)	20 (2.1)			
03:03		1 (0.1)			
15:02	2 (0.4)	2 (0.2)			
					7.79 × 10^−26^

The gene was in Hardy–Weinberg equilibrium in controls, *P* = 0.38.

* Frequencies of the four-digit subtypes are given in the Supporting Information (Table S3).

† The rare alleles, 16,10:01, 14, 03:03, 15:02, were grouped together.

‡ Odds ratios were calculated with respect to a single reference allele using logistic regression (see Statistical methods).

In Europeans, HLA-DRB1*15 confers greatest protection from Type 1 diabetes associated with the alleles HLA-DRB1*15:01 and HLA-DQB1*06:02 (see also Supporting Information, Table S1 and S2). This was not the case in our African American samples (Table 1 and 2). The HLA-DRB1*15 allele, which, despite a frequency of 0.14 in African American control subjects that is consistent with Europeans (see also Supporting Information, Table S1), was more common than expected in our African American case subjects: 0.06 compared with ≤ 0.01 in European subjects with Type 1 diabetes. However, this was not associated with the common European HLA-DRB1*15 allele, HLA-DRB1*15:01, but HLA-DRB1*15:03, which is rarely observed in Europeans (see also Supporting Information, Table S1), and had a frequency of 0.04 and 0.12 in our African American case and control subjects, respectively. Both HLA-DRB1*15:01 and HLA-DRB1*15:03 were less protective for Type 1 diabetes in our African American samples [odds ratio 0.36 (95% CI 0.11–1.11), odds ratio 0.39, 95% CI 0.19–0.80)] than HLA-DRB1*15:01 in Europeans (odds ratio 0.05, 95% CI 0.04–0.07; Supporting Information, Table S1).

In contrast to Europeans, in whom the HLA-DRB1*09:01 allele is neutral for Type 1 diabetes risk (Supporting Information, Table S1), in our African American samples, it confers susceptibility (Table 1; odds ratio 2.35, 95% CI 1.09–5.09). Lastly, at *HLA-DQB1*, HLA-DQB1*04:02 is more common than observed in Europeans (Supporting Information, Table S2).

**Table 2:** Allele frequencies and association results for *HLA-DQB1* in 225 African American cases of Type 1 diabetes and 461 controls

	Allele frequency, *n* (%)				
				
Allele	Cases	Controls	OR [95% CI]*	OR [95% CI]*	*P*_T1D_
02:01	86 (19.1)	48 (5.2)	4.25 [2.47–7.32]	1.94 [1.19–3.18]	
03:02	63 (14.0)	39 (4.2)	3.46 [1.90–6.31]	1.58 [0.93–2.71]	
02:02	92 (20.4)	115 (12.5)	2.19 [1.30–3.67]	1.00 (reference)	
03	10 (2.2)	28 (3.0)	1.24 [0.51–3.04]	0.57 [0.25–1.29]	
06	33 (7.3)	92 (10.0)	1.00 (reference)	0.46 [0.27–0.77]	
05	64 (14.2)	191 (20.7)	0.96 [0.58–1.58]	0.44 [0.29–0.67]	
03:19	25 (5.6)	82 (8.9)	0.94 [0.50–1.74]	0.43 [0.24–0.76]	
03:01	25 (5.6)	82 (8.9)	0.82 [0.44–1.52]	0.37 [0.21–0.66]	
04:02	18 (4.0)	66 (7.2)	0.79 [0.40–1.56]	0.36 [0.19–0.69]	
06:02	34 (7.6)	175 (19.0)	0.54 [0.31–0.94]	0.25 [0.15–0.40]	
02:03		4 (0.4)			
					1.62 × 10^−23^

The gene was in Hardy–Weinberg equilibrium in controls, *P* = 0.37.

* Odds ratios were calculated with respect to a single reference allele using logistic regression (see Statistical methods).

*HLA-DQA1* alleles were also very strongly associated with Type 1 diabetes in African Americans, reflecting the strong linkage disequilibrium with highly disease-associated *HLA-DRB1* and *HLA-DQB1* alleles (Table 3). However, in the haplotype analysis results section below, we do present evidence of an independent effect of *HLA-DQA1* on a specific haplotype background.

**Table 3:** Allele frequencies and association results for *HLA-DQA1* in 225 African American cases of Type 1 diabetes and 461 controls

	Allele frequency, *n* (%)				
				
Allele	Cases	Controls	Odds ratio [95% CI]‡	Odds ratio [95% CI]‡	*P*_T1D_
03:02	50 (11.1)	18 (2.0)	3.07 [1.65–5.71]	5.09 [2.97–8.74]	
05:01	88 (19.6)	57 (6.2)	2.67 [1.54–4.62]	4.43 [2.81–6.97]	
03:01	81 (18.0)	84 (9.1)	2.47 [1.44–4.25]	4.09 [2.60–6.45]	
02:01	38 (8.4)	84 (9.1)	1.00 (reference)	1.66 [1.02–2.70]	
01:01	43 (9.6)	139 (15.1)	0.72 [0.42–1.25]	1.20 [0.76–1.90]	
Rares†	15 (3.3)	46 (5.0)	0.69 [0.33–1.43]	1.14 [0.59–2.21]	
05:05	29 (6.4)	112 (12.1)	0.67 [0.37–1.22]	1.11 [0.67–1.83]	
04:01	31 (6.9)	104 (11.3)	0.67 [0.37–1.21]	1.10 [0.67–1.82]	
01:02	75 (16.7)	278 (30.2)	0.60 [0.37–0.98]	1.00 (reference)	
01:03	13 (2.9)	41 (4.4)			
05:03		1 (0.1)			
06:01	2 (0.4)	4 (0.4)			
					2.13 × 10^−23^

The gene was in Hardy–Weinberg equilibrium in controls, *P* = 0.55.

† Rare alleles comprise ^*^01:03, ^*^05:03, ^*^06:01.

‡ Odds ratios were calculated with respect to a single reference allele using logistic regression (see Statistical methods).

### Conditional analyses

Tests for additional independent signals of association in the HLA region in our African American samples showed neither rs9273363, *HLA-DQB1* nor *HLA-DQA1* added to a model with *HLA-DRB1* (*P* = 0.059, 0.089 and 0.055, respectively). All SNP associations conditional on *HLA-DRB1* were unconvincing (*P* > 1 × 10^–4^; Supporting Information, Fig. S4). The most evidence of association was at rs1383264 (*P* = 2.00 × 10^–4^) and rs3129716 (*P* = 1.50 × 10^–4^), both of which are intergenic in the class II region and so their marginal associations are probably because of linkage disequilibrium with *HLA-DQB1* and *HLA-DQA1*. We would expect the HLA-DQ genes to add to *HLA-DRB1* if we had a larger sample set, and lack of additional association signals is probably attributable to insufficient power.

### Haplotype analyses

The African-specific HLA-DRB1*03:02 allele, which is associated with protection for Type 1 diabetes, occurs on the HLA-DQA1*04:01-HLA-DQB1*04:02 haplotype and on no other HLA haplotype in our samples (Table 4). Exchange of HLA-DRB1*03:02 for HLA-DRB1*08 removes the protective effect of the HLA-DQA1*04:01-HLA-DQB1*04:02 haplotype (Table 4) despite both HLA-DQ alleles conferring protection in a single locus analysis, indicating *HLA-DRB1* is likely to be causal. HLA-DRB1*08 is more common in our African American samples (0.07) than in Europeans (0.02), which is attributable to HLA-DRB1*08 occurring on the HLA-DQA1*04:01-HLA-DQB1*03:19 haplotype, a haplotype rarely observed in Europeans. HLA-DRB1*07:01 is protective in European populations, but not in our African American samples (odds ratio 1.63, 95% CI 0.89–2.99; Table 1). However, as reported by others 5,6, we observed that the effect of the HLA-DRB1*07:01 haplotype on Type 1 diabetes risk is dependent on the *HLA-DQA1* allele present. The HLA-DRB1*07:01-HLA-DQA1*02:01-HLA-DQB1*02:02 haplotype is neutral for risk on Type 1 diabetes (frequency 7.3% in cases, 6.4% in controls). In contrast, HLA-DRB1*07:01-HLA-DQA1*03:01-HLA-DQB1*02:02, confers susceptibility to Type 1 diabetes (frequency 3.1% in cases, 0.8% in controls; odds ratio 5.28, 95% CI 1.77–15.81; Table 4). Similarly, HLA-DRB1*07-HLA-DQA1*03:02-HLA-DQB1*02:02 had a frequency of 2.0% in cases and 0.4% in controls. We also note that although HLA-DRB1*15:03 is more common than HLA-DRB1*15:01 allele, they both occur on the same haplotype background, namely, HLA-DQA1*01:02-HLA-DQB1*06:02.

**Table 4:** *HLA-DRB1*. *HLA-DQA1*. *HLA-DQB1* African American haplotypes, in 225 African American cases and 461 African American controls with complete genotypes at all three genes

Haplotype	Frequency/%		Odds ratio [95% CI]	
		
DRB1.DQA1.DQB1	Cases	Controls		
0701.0301.0202	3.1	0.8	1.93 [0.68–5.48]	5.28 [1.77–15.81]
0701.0302.0202	2.0	0.4		
04.0302.0302	4.2	0.8	1.37 [0.47–4.06]	3.75 [1.25–11.31]
0301.0501.0201	18.9	5.1	1.00 (reference)	2.73 [1.52–4.91]
08.0401.0402	2.2	0.8		
04.0301.0302	9.3	3.3	0.72 [0.37–1.37]	1.96 [0.97–3.92]
0901.0302.0202	2.7	0.3		
0901.0301.0202	2.9	1.8	0.53 [0.24–1.18]	1.44 [0.60–3.47]
0701.0201.0202	7.3	6.4	0.37 [0.20–0.66]	1.00 (reference)
13.0102.05	2.4	2.7	0.32 [0.13–0.78]	0.87 [0.35–2.17]
01.0101.05	5.6	6.2	0.29 [0.16–0.52]	0.78 [0.40–1.54]
13.0102.06	4.2	4.7	0.26 [0.14–0.50]	0.72 [0.35–1.47]
13.0505.0301	1.5	1.7	0.28 [0.09–0.87]	0.77 [0.23–2.53]
04.0301.0301	0.9	1.3		
1001.0101.05	2.2	3.1	0.20 [0.09–0.47]	0.56 [0.23–1.33]
11.0505.0319	2.4	3.9	0.22 [0.10–0.45]	0.59 [0.26–1.32]
08.0401.0319	2.9	4.0	0.21 [0.09–0.47]	0.57 [0.24–1.34]
13.0103.06	2.7	3.9	0.18 [0.07–0.41]	0.48 [0.19–1.18]
13.0201.0202	0.9	1.8		
13.0505.0319	0.3	1.0		
14.0101.05	0.7	1.7		
11.0102.05	1.1	1.4	0.14 [0.04–0.43)	0.38 [0.12–1.18]
11.0102.0602	2.0	4.5	0.13 [0.05–0.30)	0.35 [0.14–0.86]
16.0102.05	0.7	1.5		
1501.0102.0602	1.1	1.8	0.13 [0.03–0.45]	0.34 [0.09–1.27]
1503.0102.0602	4.0	11.1	0.11 [0.05–0.22]	0.30 [0.14–0.62]
0302.0401.0402	1.8	6.4	0.09 [0.04–0.22]	0.25 [0.10–0.64]
11.0505.0301	0.9	3.6	0.08 [0.03–0.23]	0.22 [0.07–0.64]
12.0101.05	0.9	3.4	0.07 [0.02–0.19]	0.19 [0.06–0.55]
Rares			0.26 [0.16–0.41]	0.70 [0.41–1.20]

The study is underpowered to detect rare effects, therefore only haplotypes with a combined frequency > 1% are listed and only those with a frequency > 1.5% are used to calculate odds ratios and 95% confidence intervals.

## Discussion

Our study of samples from donors with African ancestry is not only the largest of its kind to date in terms of number of Type 1 diabetes cases analysed for association, marker resolution and coverage of the extended MHC region, it also controls for bias from population substructure. The alleles that confer the greatest susceptibility and protection for Type 1 diabetes at the *HLA-DRB1* locus, 03:01 and 03:02, respectively, differ at four exon 2-encoded amino acids, positions 26, 28, 47 and 86 with HLA-DRB1*03:02 encoding phenylalanine, glutamic acid, tyrosine and glycine, respectively, while HLA-DRB1*03:01 encodes tyrosine, aspartic acid, phenylalanine and valine, respectively. These differences could well alter the peptide-binding specificity and capacity of these class II allotypes and their interactions with T-cell receptors, in particular the glycine to valine difference at position 86, a residue that forms part of peptide binding pocket 1 and has been associated with disease susceptibility previously and peptide binding 16,17.

The specific association of *HLA-DQA1* on the HLA-DRB1*07:01 haplotype was also confirmed in our samples (Table 4), which had more power than previous studies 5,6. In addition, owing to improved resolution of the HLA genotyping system used, and in contrast to a previous study 6, we were able to distinguish HLA-DQB1*02 subtypes and found that the HLA-DQB1*02:02 allele rather than the HLA-DQB1*02:01 allele was present on this HLA-DRB1*07:01 haplotype.

Finally, we note the unexpectedly high frequency of the protective HLA-DRB1*15 haplotypes in our African American cases compared with Europeans, does not indicate inclusion of non-autoimmune Type 1 diabetes or cases with Type 2 diabetes. The excess was attributable to the HLA-DRB1*15:03 allele, which is rarely observed in Europeans, and is less protective for Type 1 diabetes than HLA-DRB1*15:01 in Europeans (Table 1 and Supporting Information, Table S1). Although HLA-DRB1*15:03 is on the same haplotype, with HLA-DQB1*06:02, this African-specific haplotype could well have different sequences affecting class II gene expression or structure, that increase its predisposition to Type 1 diabetes. For example, the African American HLA-DRB1*15:03 allele's exon 2 sequence differs from 15:01 at amino acid, position 29 (tyrosine to histidine).

In conclusion, given the differences in risk of Type 1 diabetes associated with HLA-DRB1*03 and HLA-DRB1*08 haplotypes, our results are consistent with the assumption that *HLA-DRB1* is causal on the HLA-DRB1*03 haplotype. Furthermore, they illustrate the utility of African samples in the analysis of common disease and a need to collect and analyse much larger sample sizes. Our study, in combination with larger studies in the future, will be important for evaluating HLA and non-HLA mediated risk of Type 1 diabetes by ethnic group, which could prove informative for identifying candidates for future causal genes and the pathways involved in disease initiation and progression.

## Supporting Information

**Figure S1.** Principal component analysis of the African American samples.

**Figure S2.** Eigenvalues of the principal components plotted against principal component number.

**Figure S3.** Q-Q plot for association of the ImmunoChip single nucleotide polymorphisms.

**Figure S4.** Association with Type 1 diabetes in African Americans conditional on *HLA-DRB1* alleles.

**Table S1.** Allele frequencies in 6201 white British subjects with Type 1 diabetes and 5323 control subjects at *HLA-DRB1*.

**Table S2.** Allele frequencies in 6198 white British subjects with Type 1 diabetes and 5295 control subjects at *HLA-DQB1*.

**Table S3.** Frequencies of HLA-DRB1*04 subtypes in African American subjects with Type 1 diabetes and control subjects.
