# Supplementary material for: HLA class II gene associations in African American Type 1 diabetes reveal a protective HLA-DRB1*03 haplotype
Source: Diabet Med. 2013 Mar 21;30(6):710–6. doi: 10.1111/dme.12148 (PMC3709123; doi:10.1111/dme.12148)
Supplement: Supplementary file 1 [file dme0030-0710-SD1.docx]

**Supplementary Information**

**Supplementary Figure 1** Principal component Analysis of the African American samples. Red are the African American Controls, Pink are the African American cases; Green are the HapMap CEPH samples; Purple are the HapMap YRI samples; Blue and yellow are the HapMap CHB and JPT samples, respectively. **A.** Are all African American cases and controls. **B**. Has the ancestral outliers removed.


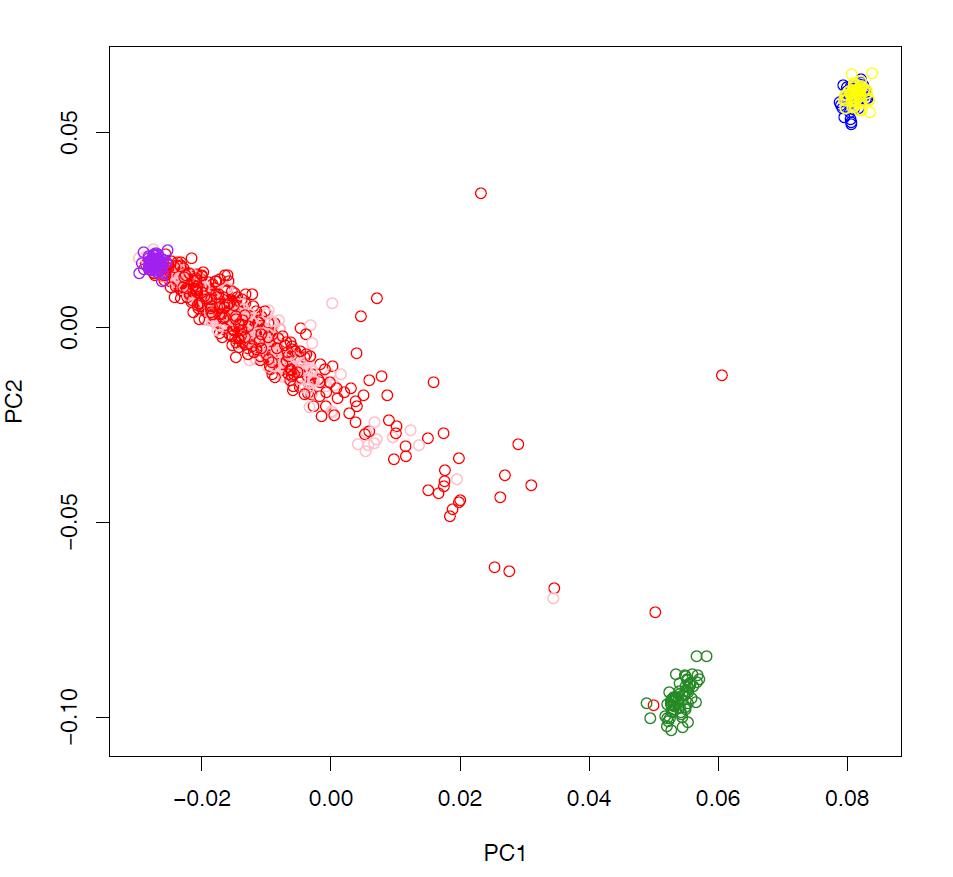


**A**


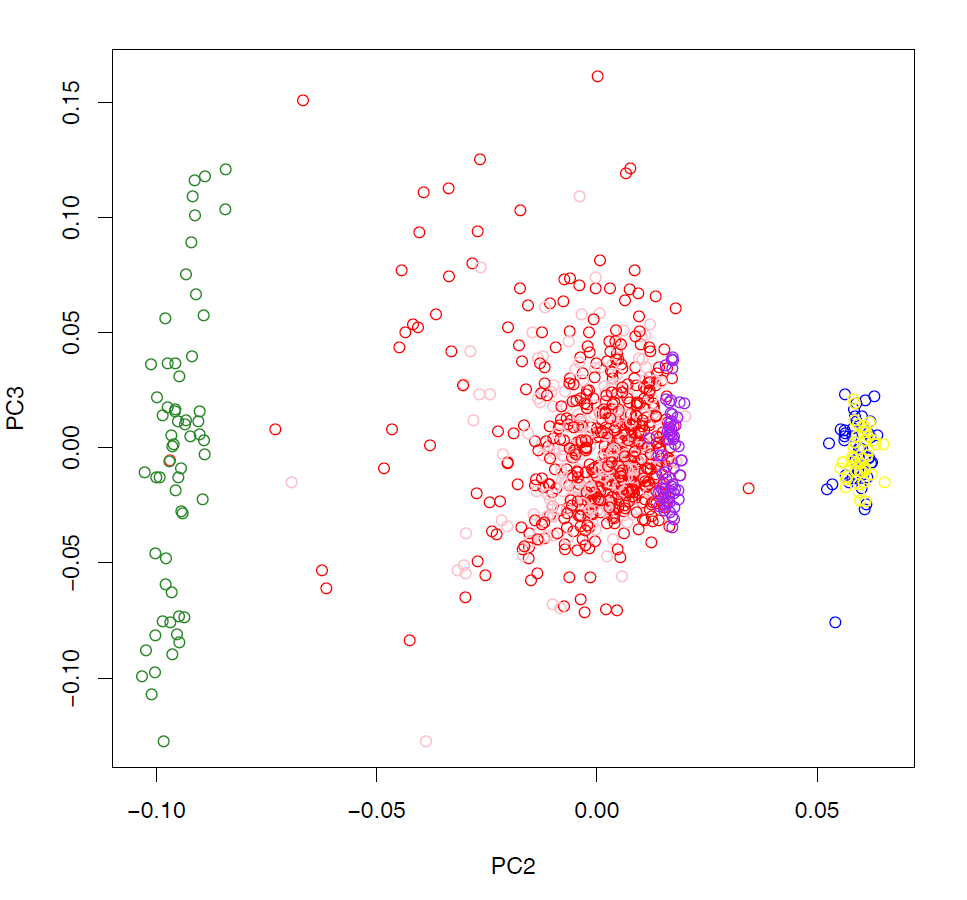


**A**


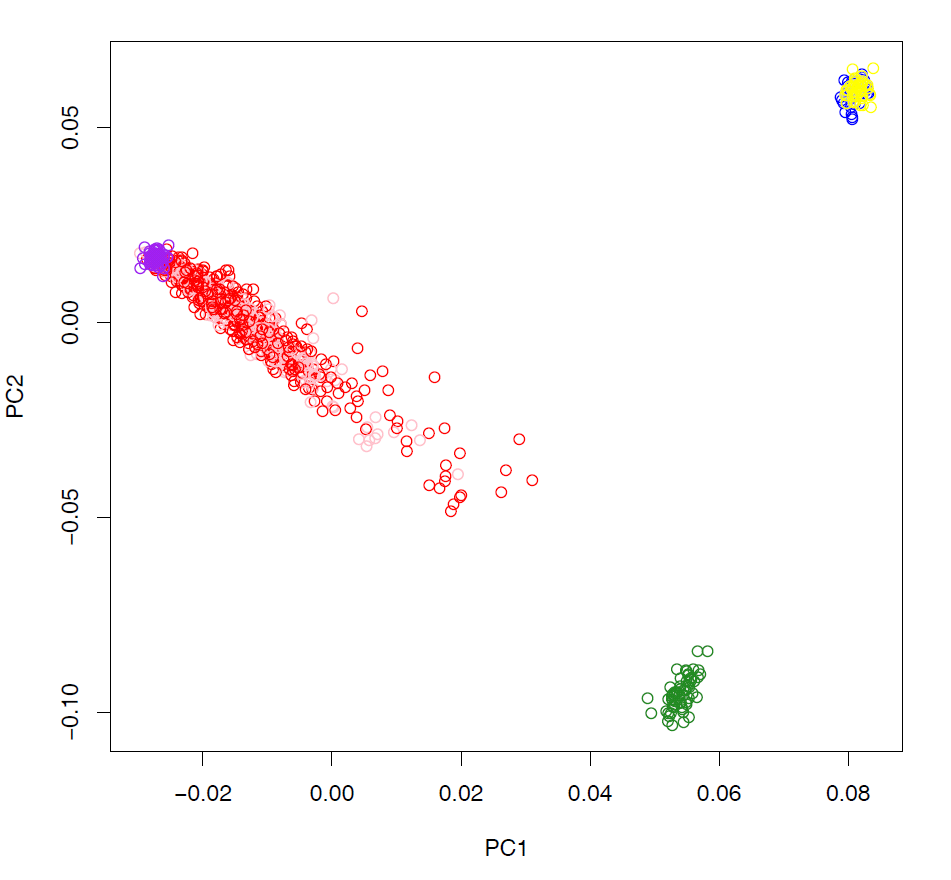


**B**


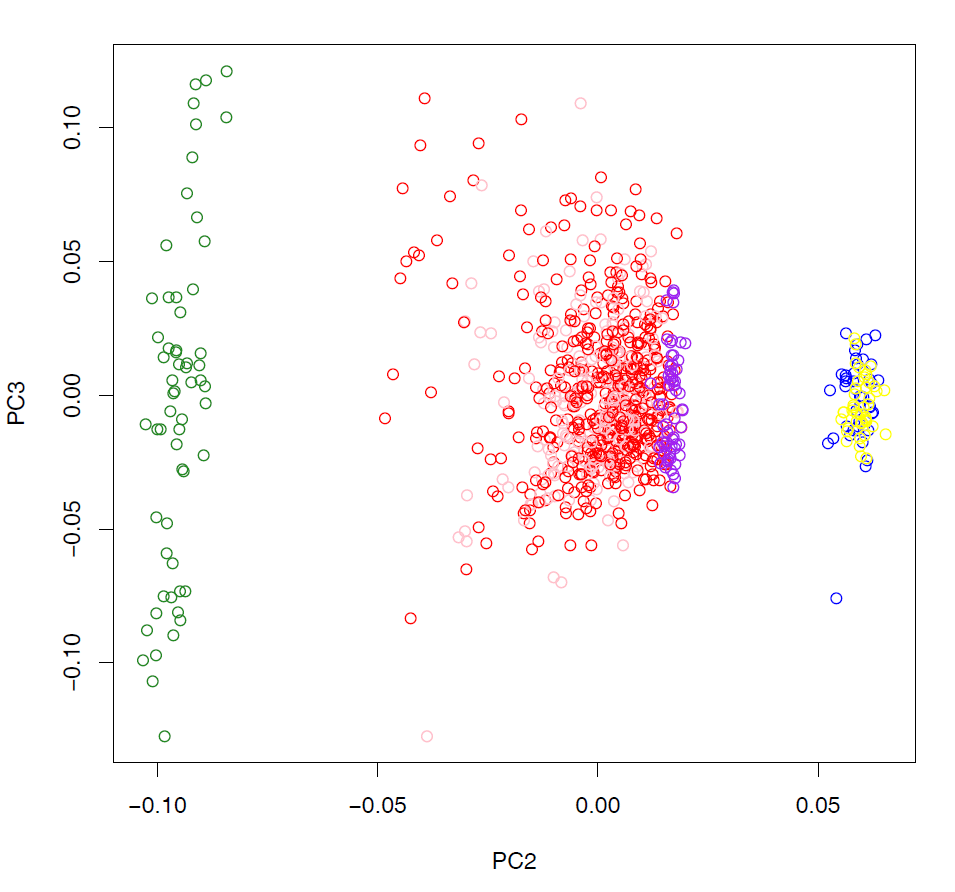


**B**

**Supplementary Figure 2** Eigenvalues of the principal components plotted against principal component number.


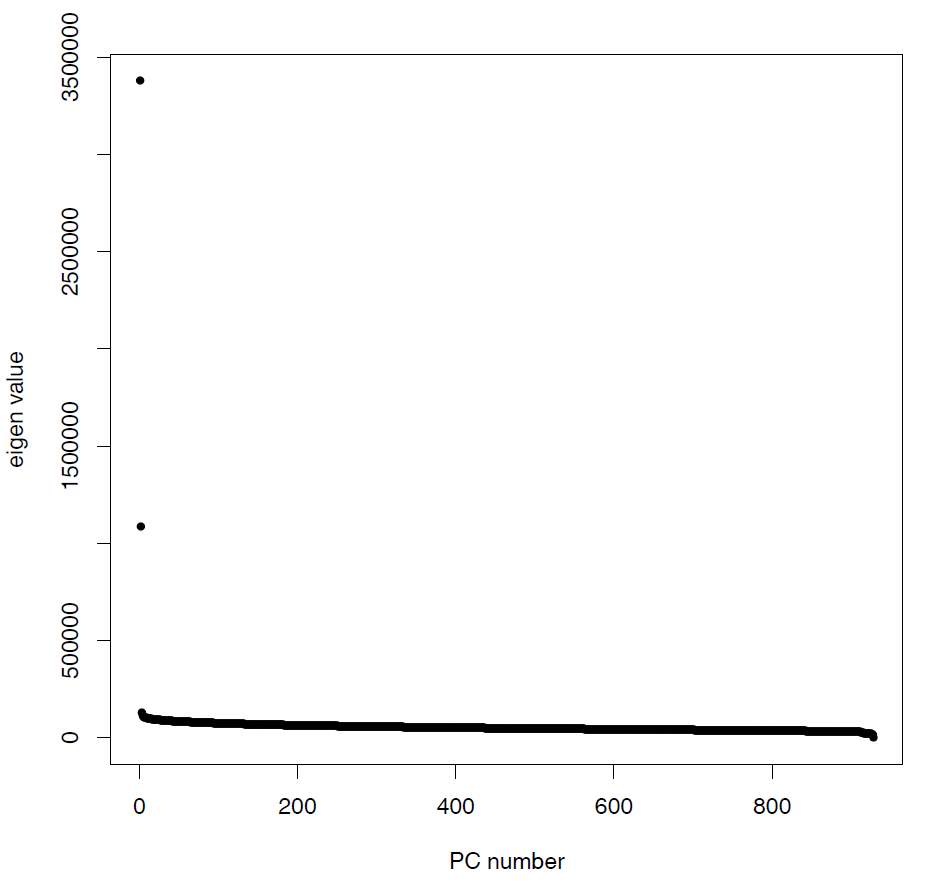


**Supplementary Figure 3** Q-Q plot for association of the ImmunoChip SNPs. **A** includes all SNPs on the chip. **B** includes only SNPs outside of the known T1D regions. Inflation of the test statistics across the ImmunoChip is expected to be elevated compared to a GWA SNP chip as the ImmunoChip is enriched for autoimmune associated SNPs, with higher SNP density in the 186 regions than on a GWAS chip. Removal of the T1D associated regions (and SNPs in strong pairwise LD with each other) results in a reduction in inflation from 11% to 3%. Dependence of test statistic inflation on SNP call rate was also considered, however raising the SNP call rate (>0.99) made no detectable difference to the observed inflation (data not shown). We anticipate that this is an acceptable level of inflation and does not indicate genotyping quality issues.

**A**


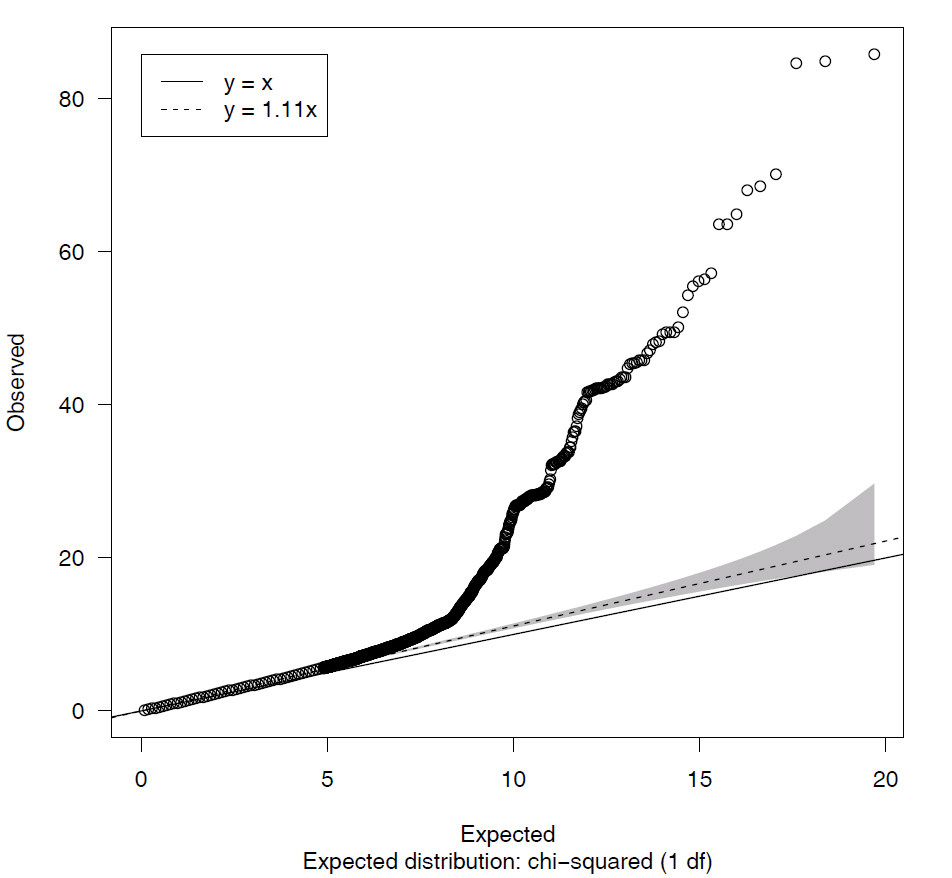


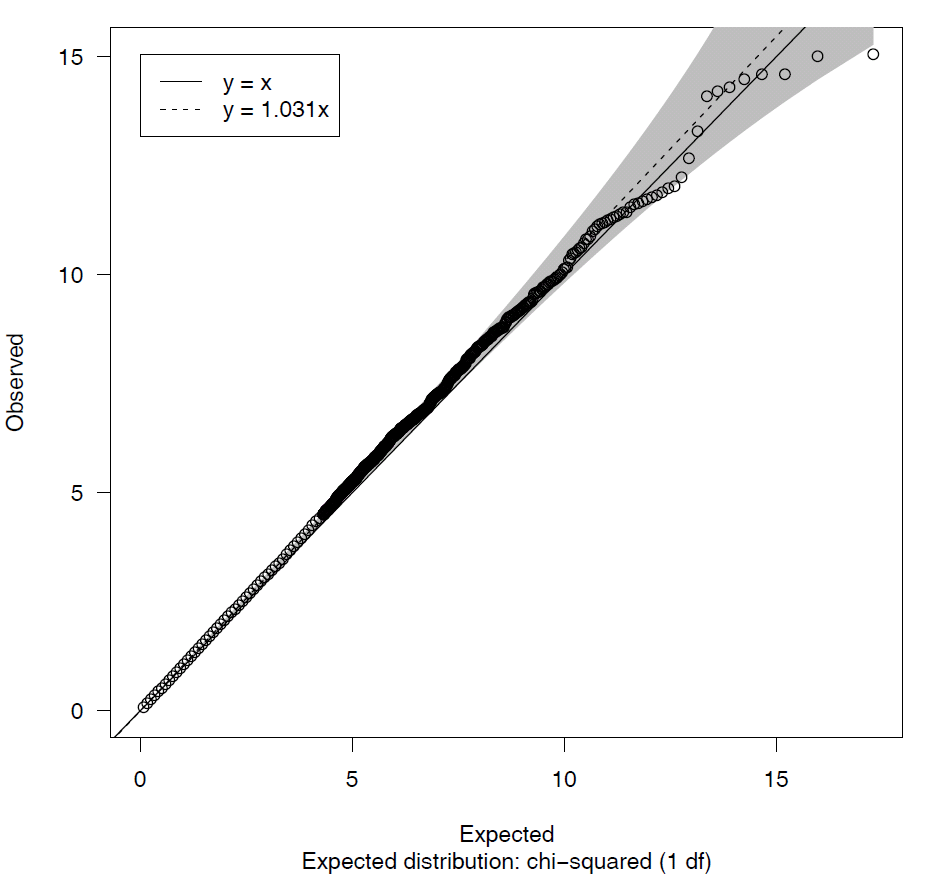


**B**

**Supplementary Figure 4** Association with type 1 diabetes in African Americans conditional on *HLA-DRB1* alleles. All analyses were adjusted for population ancestry using the first three principal components.





**Supplementary Information – HLA class II association in white British samples**

For ease of comparison with our analyses in African American samples, below we report allele frequencies and effect sizes for the class II alleles in British type 1 diabetes cases and controls. These collections have been described elsewhere (2; 11; 15). In brief, the cases and controls are matched for geographical origin within Great Britain and sex. Cases were diagnosed with type 1 diabetes before age 17 years, with a mean age-at-diagnosis of 7.6 years. These are the data analysed in reference 15, however, note, that although allelic odds ratios are reported below, the true mode of inheritance is not multiplicative and so, these are only reported for comparison with our African American associations. Genotype effects are reported in reference 15. In the African American samples, no deviation from multiplicative allelic effects on the odds ratio scale was detected, however this is likely to be attributable to lack of power.

**Supplementary Table 1** Allele frequencies in 6,201 white British type 1 diabetes cases and 5,323 controls at *HLA-DRB1* [15]. Rare protective alleles (<0.01 frequency) are grouped together to calculate odds ratios (OR) and 95% confidence intervals (CI).

| **Allele** | **Cases**  **N (freq)** | **Controls**  **N (freq)** | **OR [95% CI]** |
| --- | --- | --- | --- |
| 04 | 5,014 (0.38) | 1,871 (0.18) | 3.60 [3.19-4.05] |
| 03:01 | 4,183 (0.34) | 1,541 (0.14) | 3.04 [2.71-3.41] |
| 08 | 309 (0.02) | 229 (0.02) | 1.69 [1.36-2.11] |
| 09:01 | 172 (0.01) | 134 (0.01) | 1.51 [1.15-1.99] |
| 01 | 1,158 (0.09) | 1,288 (0.12) | 1.00 (reference) |
| 13 | 555 (0.04) | 1,035 (0.10) | 0.57 [0.49-0.66] |
| 07:01 | 582 (0.05) | 1,568 (0.15) | 0.43 [0.38-0.50] |
| Rares |  |  | 0.33 [0.27-0.41] |
| 14 | 17 (0.001) | 247 (0.02) | NA |
| 12 | 58 (0.005) | 158 (0.01) | 0.40 [0.28-0.56] |
| 16 | 67 (0.005) | 69 (0.01) | NA |
| 04:03 | 39 (0.003) | 131 (0.01) | NA |
| 04:07 | 14 (0.001) | 82 (0.01) | NA |
| 10:01 | 13 (0.001) | 57 (0.01) | NA |
| 11 | 149 (0.01) | 691 (0.06) | 0.24 [0.20-0.30] |
| 15:01 | 63 (0.005) | 1,514 (0.14) | 0.05 [0.04-0.07] |
| 15:02 | 9 (0.001) | 31 (0.003) | NA |

**Supplementary Table 2** Allele frequencies in 6,198 white British type 1 diabetes cases and 5,295 controls at *HLA-DQB1* [15].

| **Allele** | **Cases**  **N (freq)** | **Controls**  **N (freq)** | **OR [95% CI]** | **OR [95% CI]** |
| --- | --- | --- | --- | --- |
| 03:02 | 4,408 (0.36) | 1,100 (0.10) | 9.10 [7.84-10.55] | 2.77 [2.51-3.06] |
| ^*^02 | 4,765 (0.38) | 2,633 (0.25) | 3.28 [2.87-3.75] | 1.00 [reference] |
| 04:02 | 299 (0.02) | 218 (0.02) | 2.38 [1.89-3.01] | 0.73 [0.59-0.89] |
| 05 | 1,252 (0.10) | 1,606 (0.15) | 1.59 [1.37-1.84] | 0.48 [0.44-0.54] |
| 06 | 536 (0.04) | 995 (0.09) | 1.00 [reference] | 0.30 [0.27-0.35] |
| 03:01 | 831 (0.07) | 1,971 (0.19) | 0.95 [0.82-1.10] | 0.29 [0.26-0.32] |
| ^†^03 | 247 (0.02) | 615 (0.06) | 0.73 [0.59-0.89] | 0.22 [0.19-0.26] |
| 06:02 | 58 (0.005) | 1,452 (0.14) | 0.08 [0.06-0.11] | 0.03 [0.02-0.03] |

^†^ Codes for 03:03 and 03:04 ^*^ Note that *HLA-DQB1* was genotyped using Dynal technology in these samples such that HLA-DQB1*02:01 and HLA-DQB1*02:02 could not be distinguished.

**Supplementary Table 3** Frequencies of HLA-DRB1*04 subtypes in African American type 1 diabetes cases and controls. One case was either a *04:01 or *04:08, but which of these two four digit subtype could not be established so is not included in the subtypes below.

| **HLA-DRB1 allele** | **Allele frequency, N (%)** | |
| --- | --- | --- |
|  | **Cases** | **Controls** |
| 04:01 | 31 (6.8) | 25 (2.7) |
| 04:02 |  | 1 (0.1) |
| 04:03 | 1 (0.2) |  |
| 04:04 | 11 (2.4) | 4 (0.4) |
| 04:05 | 29 (6.3) | 18 (1.9) |
| 04:07 | 1 (0.2) | 8 (0.8) |
| 04:08 |  | 1 (0.1) |
| 04:35 |  | 1 (0.1) |
